# Two Step Excitation in Hot Atomic Sodium Vapor

**DOI:** 10.1038/s41598-017-12089-w

**Published:** 2017-09-18

**Authors:** Bernd Docters, Jörg Wrachtrup, Ilja Gerhardt

**Affiliations:** 1 3rd Institute of Physics, University of Stuttgart and Center for Integrated Quantum Science and Technology, IQST, Pfaffenwaldring 57, D-70569 Stuttgart, Germany; 2 Max Planck Institute for Solid State Research, Heisenbergstraße 1, D-70569 Stuttgart, Germany

## Abstract

A two step excitation scheme in hot atomic sodium vapor is experimentally investigated. The observed effects reflect a coupling between the 3^2^S, 3^2^P and the 3^2^D states. We present the relative dependence on detuning of the two utilized lasers around λ = 589 nm and 819 nm. Unlike expected, we achieve a higher detuning dependence of the probe and the coupling laser by a factor of approximately three. The presented work aimed for a Rydberg excitation and quantum light storage. Such schemes are usually implemented with a red laser on the D-line transition and a coupling laser of shorter (typically blue) wavelength. Due to the fact that higher P-Rydberg states are approximately two times higher in energy than the 3^2^D state, a two photon transition from the atomic excited 3^2^P state to a Rydberg P state is feasible. This might circumvent laser frequency doubling whereby only two lasers might mediate a three photon process. The scheme of adding three *k*-vectors allows for electromagnetically induced transparency experiments in which the resulting k-vector can be effectively reduced to zero. By measurements utilizing electric fields and an analysis of the emission spectrum of the atomic vapor, we can exclude the excitation of the P-P two photon transition.

## Introduction

Atomic vapors allow for highly coherent spectroscopic experiments, which are even accessible in Doppler broadened hot atomic vapor cells^1^. A particularly interesting version is the observation of electromagnetically induced transparency (EIT)^2–4^. In a variety of experimental schemes, a coupling with two laser fields forms a coherence between the two hyper fine transitions in an atomic medium called a so-called lambda-scheme, although other schemes have also been reported in the literature^5^.

An interesting feature of EIT, is the option to store photonic states in the atomic medium^6–8^. Such quantum memories for light can be implemented down to the single photon level^9^. A storage of single photons, e.g. from a down conversion source, or from a single emitter has not yet been implemented due to the large bandwidth of the commonly available single photon sources. Presently, several groups are working world-wide towards this goal, since this is one of the requisite features for future quantum information and quantum communication schemes. An interesting option would be to use of single photons originating from a single molecule^10^, which can be simultaneously be both extremely bright and narrow-band. These photons show near-unity Hong-Ou-Mandel indistinguishability and exhibit no further background from e.g. other impurities upon optical filtering via an atomic Faraday filter^11,12^.

Since the turn of the 21st century, multilevel step-wise (ladder scheme) excitations towards Rydberg states in atomic vapors, have garnered much attention. These equivalently allow for high coherence, between an alkali P state and a higher order state^13,14^. These schemes are commonly described as Rydberg-EIT. Instead of the coherence into another ground state, the coherence is implemented between a ground state and a (long-lived) Rydberg state. Usually EIT in the ladder scheme utilizing hot atomic vapors is observed in a configuration of a longer-wavelength ground-state (probe-)laser, and a shorter wavelength (coupling-)laser^15,16^. Otherwise, the signal is strongly suppressed by the Doppler broadened medium. These schemes allow for a storage and retrieval of laser pulses^17^. Due to the limited spatial extension of the so-called spin-wave, such experiments are to-date limited to cold atomic vapors.

Research on atomic sodium vapor has lost the attention of the scientific community over the years, since the heavier alkali atoms have a more convenient wavelength and exhibit nicely split D-line transitions. Moreover, sodium requires a higher operating temperature and tends to diffuse into the glass of vapor cells, further augmenting the difficulties of experimentation. Few experiments on atomic sodium with ladder scheme excitations have been performed. These do not necessarily aim for the observation of EIT, but aim for experimental investigations of four-wave mixing^18^, conical emission^19^, and other ladder type transitions^20–22^. One spectroscopic feature is the often observed transition between the 3^2^P_3/2_ and the 3^2^D_5/2_ state in sodium, which is located in the near-infrared, around 819 nm. This line can also be observed in lighting applications and represents the strongest spurious line of high pressure sodium vapor lamps^23^. In preparation to experiments involving a Rydberg excitation of hot atomic sodium vapor, we perform experiments in which the hot gas is excited with the common D-line transitions around 589 nm and additionally around 819 nm. This transition is approximately mid-way between the 3^2^P state and the Rydberg states close to the ionization threshold.

For quantum storage experiments, the spin wave extension, whose length is determined by a joint relative phase in the atomic excitation by both laser fields, is important, and is commonly very short in Rydberg EIT schemes. This originates from the large wavelength mismatch, and limits their applicability to the above mentioned storage schemes. Therefore, clever experiments were envisioned and performed in which three incoming waves were overlapped, such that their *k*-vectors add up to zero^24,25^ and the excitation is effectively Doppler-free. This subsequently allows for a very large effective spin wave at the point of excitation. Experimentally, such schemes were implemented in the past^26^. These require the careful alignment and the suppression of aberrations in all three beams such that the effective *k*-vector is reduced to zero. At higher excitation powers, other effects such as self-focusing might come into play, which then require again a different alignment^19^.

**Figure 1:**
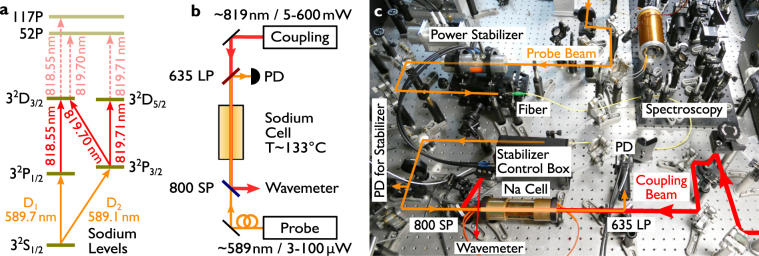
Level Scheme and Experimental Setup. (**a**) the involved atomic levels as derived from the NIST database^ 29^. The excitation from the 3^2^S → 3^2^P state is probed, whereas the 819 nm laser should couple the 3^2^P state to a higher P state. The intermediate D state should help for the excitation into the higher state. (**b**) experimental configuration. Two counter propagating lasers excite the sodium atoms in the atomic vapor. To note is that the infrared laser is used in an extremely high power regime. (**c**) Photograph of the experimental setup. To note: To increase the probe beam quality, the light is mode-filtered by an optical fiber. The 819 nm infrared-laser is directly supplied from the Ti:Sa laser.

As reported for rubidium and cesium in the past^13,14^, our set of experiments aims to observe Rydberg-EIT in a hot atomic vapor of sodium. Instead of frequency doubling infrared light as typical in such experiments, we envision driving an (allowed) two photon transition between the 3^2^P and a higher Rydberg P state via an almost resonant D state. Other two photon transitions are highly topical in this line of research, as has been the case with atomic rubidium vapor^27,28^. The mentioned 3^2^D states lie by chance somewhere midways in between these allowed two photon transition and would facilitate the excitation into a higher Rydberg state as discussed below. The envisioned excitation scheme is presented in Fig. 1a. The spectroscopy on the 3^2^D states is well reported in the literature, although still with very old spectroscopic data, the higher Rydberg states can only be estimated by somewhat outdated quantum defects^29^.

Here we present our experimental attempts to drive a three photon ladder scheme excitation with only two lasers. The *probe* laser drives the 3^2^S → 3^2^P transitions (the sodium D-lines) continuously. The second much stronger (*coupling-*)laser, with a few hundred milliwatts of optical power, is close to resonance with the 3^2^P → 3^2^D transitions, but might also excite a two photon excitation from the 3^2^P states into either the 52^2^P state, from the sodium D_2_ line or, alternatively, the 117^2^P state from the sodium D^1^ line. An estimation of the two photon coupling strength is presented below.

## Setup and Data Acquisition

The ladder scheme excitation is depicted in Fig. 1a. A probe laser (589 nm) is scanned over the sodium D_1_- or the D_2_-line. In sodium both D-lines are separated by approximately 0.6 nm (515.52 GHz). A second, counter-propagting infrared laser couples the atomic population with the higher 3^2^D state. The same laser might be suitable to address a higher P-Rydberg state. For the appropriate selection of velocity classes in the hot atomic vapor, a counter-propagating laser excitation is required. This corresponds to a calculation of accounting for the velocity classes in the Doppler broadened medium, and the selection of involved wavelength^15,16^.

Figure 1b shows a scheme of the experimental setup. The two counter-propagating laser beams are aligned collinearly. The probe beam (589 nm) is reflected off a 650 nm long pass filter (650 LP, Fig. 1b) and guided to a large area photo detector. The filter was introduced to enable the (counter-propagating) infrared beam to pass through the cell. When the coupling laser is well aligned, the infrared light leaks through the single mode fiber, which is used for mode cleaning the probe laser.

The sodium vapor cells were produced with on-demand specifications by the glass workshop in house (see acknowledgments). The dimensions of the cells amount to 100 mm optical length and 26 mm inner diameter. The cells are heated by resistive heaters around the cell windows to prevent the formation of a metal mirror on them. The rest of the cell is thermally shielded from the environment by a glass cylinder, slipped over the heated copper blocks, which are heated with electrical heaters at the end of the glass vapor cell. The temperature was stabilized over approximately one day before commencing the experiment to a temperature of T = 133–134 °C, which corresponds to approximately 5.3 ×10^10^ atoms/cm^3^ ^30^.

The probe laser is delivered by a dye ring laser system (899–29, Coherent), running on rhodamine 6 G. The laser frequency is scanned in the course of the experiment with the available laser control box over a range around 8–10 GHz. The spectroscopic alignment to the sodium D-lines is performed with a spectroscopy setup (Fig. 1c, top, right), which features the following signals: a) Doppler free spectroscopy, b) frequency modulation spectroscopy (using an electro optical modulator, Fa. Qubig, modulation frequency 9 MHz) and c) modulation transfer spectroscopy^31^. This allows for a comprehensive set of fine resolved reference signals for all laser frequency scans.

For the data presented here, the probe laser is scanned and an according transmission spectrum is recorded. Intensity fluctuations, e.g. caused by a spectral detuning of the laser’s ring cavity, would present problems to this procedure. Therefore, we stabilized the intensity of the scanning (probe-)laser with a commercial “noise eater” solution (Conoptics). The electro optic modulator of the device is placed in the beam leaving the laser, whereas the detector of the stabilizer is placed behind an optical single mode fiber (Nufern 460-HP, and a Thorlabs/Geltech C220-TME-A out coupling lens), used for mode cleaning the beam before it is transferred to the atomic vapor cell. The “noise eater” is able to suppress the noise level of the probe beam well below the 1% range for low frequencies (1–100 Hz).

The coupling beam was generated by a Titanium:Sapphire laser (Ti:Sa, 899-21 ring laser from Coherent inc.). The laser’s line width amounts, according to the laser’s manual, to 500 kHz-1 MHz, similar to the dye ring laser. The beam is used as it emerges from the laser system without any further beam-shaping or mode clean up. The laser system exhibits a focal width of *w*_0_ = 300 μm. This waist is, according to the laser’s manual, located 50 mm from the outcoupling mirror inside the laser. The vapor cell center is located at a position of approximately 870±5 mm behind the laser output extension (used to transmit a part of the beam to a reference cavity). The beam radius of the beam which is later entering the cell is measured at five positions along the beam path. Error propagation for each measurement results in a standard deviation of the fits of maximally 4 μm for each measurement. Gaussian beam calculations (using the equation $w(z)={w}_{0}\,\sqrt{1+{(\frac{z}{{z}_{{\rm{R}}}})}^{2}}$ with ${z}_{{\rm{R}}}=\frac{\pi {w}_{0}^{2}}{\lambda }$) reveal the beam waist to be 910 ± 7 μm at the input of the cell, and 992 ± 7 μm at the end of the cell. Therefore, the area of the beam is increased from the front of the cell to the back of it by 19%. For the calculations of the Rabi frequencies below, the beam waist is assumed to be 950 μm. The exact frequency of the Ti:Sa laser is measured with a wavemeter (Coherent Wavemaster) with an uncertainty of ±0.005 nm (value from the data sheet, approximately 5 GHz (or 0.1–0.2 cm^−1^) in the 819 nm spectral region). For this measurement, a part of the beam was sampled out right before the beam dump of the infrared laser (see Fig. 1b).

The beam diameters are adapted such that the probe beam is significantly smaller than the coupling beam to fully transfer the atomic population to a higher state. The probe beam diameter, 2× *w*0, amounts to 120 ± 7 μm at the center of the vapor cell, whereas at the ends of the cell the diameter is determined as 330 ± 7 μm. This beam diameter has been determined by a Gaussian beam calculation and subsequent measurements by a razor blade, comparable to the measurements of the coupling laser beam. We estimate the uncertainty to be comparable to the coupling beam (above). Both beams have been course aligned with implemented irises in the setup, and later with the aid of the single mode fiber of the probe beam. We additionally experience a strong alignment dependence of the derived signals to the coupling beam. To avoid an influence on the intensity stabilization of the probe laser by the 819 nm coupling beam, a 800 nm short pass filter (800 SP in Fig. 1b) was introduced which reflects the beam towards a wavemeter.

The laser power for the experiments presented below are recorded with two commercial laser power meters (Thorlabs, PM200 with a S130C sensor; Coherent field master). The power meter reading is averaged for about a second and the raw reading is presented with the results below.

The signal of the probe beam was recorded with an amplified photo diode (Thorlabs, PDA-36A-EC). The polarization of the beams involved has been fixed by a polarizing beam splitter on the optical path of the probe beam. The pump beam’s polarization is unchanged from the laser’s polarization. In summary, both beams are linearly polarized and are orthogonal to one another in the vapor cell. To suppress reflections from the setup back into the laser cavity, we added an optical isolator just right behind the output coupler of the Ti:Sa laser. It is worth mentioning that this did not change the acquired signals, although the exciting polarization was turned by up to 45°.

All relevant signals were recorded with an oscilloscope (LeCroy, Wavepro 7k) via an automated acquisition procedure over a network connection. The spectral position of the infrared laser was controlled by the voltage output of a measurement card (National Instruments, USB-6361). Several frequency scans of the probe laser were recorded and accumulated, which was automatically scanned by the laser control box (approximately 250 ms per 8–10 GHz); following this, the infrared laser was detuned, and another cycling of the probe laser was performed to amount to a 2D scan. The recorded channels were a) the trigger signal from the dye laser control box, b) the FM-spectroscopy signal, c) the photo diode for the probe laser.

A photograph of the experimental setup is presented in Fig. 1c. It shows the atomic cell in the front and the probe beam passing the cell from the left of the picture. On the right hand side, the 899 Ti:Sa laser and an optical isolator is placed (not shown).

## Results

The first set of experiments are a recording of the (usual) absorption spectrum of the hot atomic sodium vapor as a reference. The transmission value for a far detuned laser was set as 100% reference. When the laser was blocked, the recording determined the 0% point. The probe power was reduced to almost zero to prevent saturation effects in the vapor. Then, the probe power was increased and it was ensured that no further changes in the spectrum were evident. With this power the following experiments are performed. In the course of the experiment, we partially exceeded the saturation intensity^30^ by a factor up to 10 (4 times D_1_-line, or 10 times D_2_-line) to achieve a reasonable high signal on the photo diode. The recorded spectrum allowed us to fine-tune the temperature, which was set to result in an absorption spectrum with 80–95% absorption. Due to the (approximately) exponential rise of the atomic vapor density with the temperature, and Beer-Lambert’s law, a slight change in temperature results quickly in a significantly higher absorption. The following experiments were all recorded around 133–134 °C. The spectra without the coupling beam are fitted against a theory model (see e.g. Refs^11,12^ and references therein) which confirmed the temperature around 133–134 °C.

The cell was slightly tilted against the optical path to exclude any artifacts by reflections from the cell windows. This is to exclude Doppler free configurations or that back-reflections are detected on the probe detector. From the side, and with several tens of μW probe power, the additional reflections within the cell are clearly visible by eye. They are set such that they are not exiting the cell in the direction of the detector. The heating configuration with the copper blocks also features a smaller beam entrance hole, such that some reflected beams were captured by them. Small alterations on the (hot) cell in the working configuration did not alter the observed signals.

**Figure 2:**
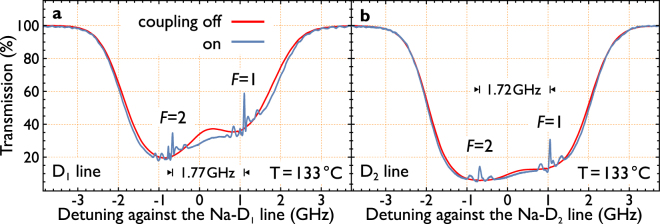
A single recorded spectrum. Transmission spectra of the sodium cell with the coupling laser turned on (blue) and turned off (red). The peaks due to enhanced transparency and dips due to enhanced absorption are clearly visible. Observed are two sets of features separated by the ground state splitting of sodium. The spectra are horizontal cuts from the density plots in Fig. 4 at zero detuning of the coupling laser. The background spectrum is obtained by averaging the transmission signals across all recorded detunings of the coupling laser. (**a**) Shows the recording for the D_1_-line. The probe power is 15 μW; the coupling laser power amounts to 440 mW. (**b**) The same recording for the D_2_-line. To note that the absorption is increased due to the higher oscillator strength of the D_2_-line. Probe power: 35 μW and coupling power of 530 mW.

When the coupling laser is brought into resonance around the 3^2^D levels, a multi feature signal appears in the continuously recorded probe spectrum. We clearly observe sub-Doppler enhanced transmission peaks, comparable to the features observed in EIT spectroscopy. This is depicted in Fig. 2. The red plotted spectrum represents an average spectrum of all lines in the course of detuning the infrared laser. The spectrum of the D_1_-line displays more features than the spectrum with the D_2_-line. This results from the higher separation of the hyper fine structure in the 3^2^P_1/2_ state (188 MHz hyper fine splitting). Otherwise, for the D_2_-line, the features are blurred out. All spectra below are presented as the difference of the average signal (Fig. 2, red) for all lines and each specific line. Then, the dominating absorption feature is suppressed and the relative height of the coupling laser influence can be determined. The measured signal depth is accounted for as a fraction of the overall absorption feature. This implies, that the measured signal strength in the D_1_ and the D_2_-feature is comparable, although the normal absorption spectrum differs as described above and visible in Fig. 2.

We now compare the laser frequency which leads to the observed features to the values of the 3^2^P_1/2_ → 3 ^2^D_3/2_ in the the NIST database^29^. Since the features do not vanish when the coupling laser is detuned, the reference point is determined, when the relative signal strength is the largest. The literature value for the 3^2^P_1/2_→ 3^2^D_3/2_ transition is given at 12216.7211 cm^−1^. In our study we observe the enhanced transmission at 12216.6 cm^−1^. Correspondingly, for the 3^2^P_3/2_ → 3^2^D_5/2_ the NIST value amounts to 12199.5 cm^−1^, which is reconfirmed by our experimental value (12199.5 cm^−1^). We like to remark that our presented values have a significantly higher uncertainty than the NIST value, since they are limited by the utilized wavemeter to approximately 5 GHz (or 0.1–0.2 cm^−1^) in the 819 nm spectral region.

More information on the origin might be derived by two dimensional spectroscopy. By extracting the relative detuning slopes one might extract how many photons are involved for the transitions. A calculation for the expected relative detuning is presented below.

**Figure 3:**
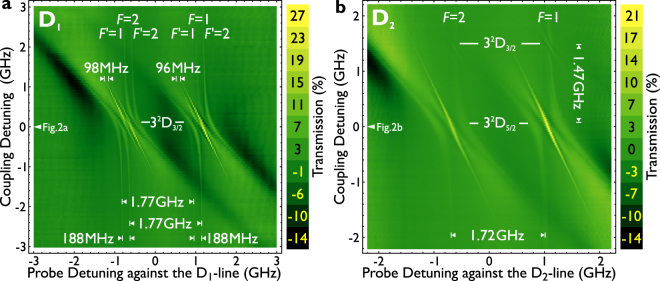
Density Plots of the D_1_ and D_2_ line. Density plots of the transparency features depending on the detunings of both lasers. The data is obtained by subtracting the background spectrum (coupling laser turned off) from the spectrum where the coupling laser is turned on (subtracting the two curves in Fig. 2 at each frequency of the coupling laser). Darker colors refer to high absorption and brighter colors refer to high transmission through the cell. The plots show the main transparency peaks moving along a straight line. The slope amounts to −2.08 ± 0.04 GHz_819 nm_/GHz_589 nm_. The observed transparencies beside the main peaks form anti crossings which are even observed at very far detuning of the coupling laser from the resonance point. Enhanced absorption (black areas) are also observed for far detunings of the lasers. In case of the sodium D_2_ line weak additional transparency features are observed, approximately +1.47 GHz apart from the main feature. The features are labeled in terms of their originating levels. The enhanced transparency and absorption values in percent are obtained by comparing the deviation of the transmission spectrum with the background spectrum (the two curves in Fig. 2), normalized by the maximum absorption of the background spectrum. For this recording the same probe and coupling laser powers as in Fig. 2 were used. Interestingly, the slope of the observed feature on the 3^2^D_3/2_−level amounts to −1.53±0.08 GHz_819 nm_/GHz_589 nm_.

Figure 3a shows the two dimensional spectrum of the detuning around the sodium D_1_-line. An arrow indicates the setting for the recording in Fig. 2. The signal presented here is the difference between each line and the average spectrum of the entire recording. We now turn to analyze the spectral features originating from the *F* = 2 ground state (left feature). On resonance, the feature is symmetric and displays six main lines of transmission enhancement. Another smaller feature (7th line) is visible between the inner most lines, which is ignored in the following. The four outer features (two on the right and two on the left) are formed from the top and from the bottom of the recording. Far from the resonance the features are separated as the splitting in the 3^2^P_1/2_ state, by 188 MHz. We like to note, that we carefully ensured that this feature is not visible at larger (tens of GHz) detuning of the 819 nm laser. This is therefore not a Doppler-free type absorption signal of the 589 nm laser alone, as the cell and other interfaces are strongly tilted against the beam. The feature further vanishes when the coupling beam (819 nm laser) is blocked. This is once more underlined by the fact, that the features are shifted against each other: The signal at negative detuning of the infrared laser is at lower energies, whereas at larger coupling detuning, the feature is also at higher frequencies for the probe laser. The two inner features are dominant at small coupling detunings and are split by approximately 98 MHz. With the detuning of the infrared laser, we observe a relative (detuning) slope of −2.08 ± 0.04 GHz_819 nm_/GHz_589 nm_. This value is determined by extracting the peaks from Fig. 3a, and fitting them with a linear regression (least squares). In practical terms this means that when the 819 nm coupling laser frequency is increased by 1 GHz, it is required to reduce the frequency of the 589 nm probe laser by 2.08 GHz to observe a similar feature in the spectrum. With the infrared laser detuning, they change their relative strength from higher to lower frequencies for the *F* = 2 level and vice versa for the *F* = 1 ground state.

Figure 3b shows the features of the same recording as before, but for the sodium D_2_ line. For this, the transitions between the hyper fine levels of the ground (3^2^S_1/2_) state *F* = 1 and *F* = 2 and the respectively excited hyper fine levels of the 3^2^P_3/2_ state *F′* = 0, 1, 2 and *F′* = 1, 2, 3 cannot be resolved completely. The transparency peaks are considered to be overlapped. The maximum of the resulting peak is called the center of mass (COM) of the transition group. Additionally to the three allowed transitions in each transition group, there are also three crossover resonances to take into account. As the zero-frequency reference, the weighted COM of the six transitions between the hyper fine levels is used. For the COM’s of the transition groups for *F* = 1 and *F* = 2 is respectively calculated 1.0669 GHz and −0.6488 GHz against the center of mass of the atomic transition, which results in a splitting of 1.7157 GHz for the observed peaks. These values are adapted from Ref.^32^. These “blurred-out” features do not allow to resolve the individual levels in the 3^2^P_3/2_ state, but as in the D_1_-line the lines are visible far detuned from the resonance. The inner feature exhibits the same slope as reported for the D_1_-line: −2.08±0.03 GHz_819 nm_/GHz_589nm_. Unlike there, the relative intensity changes from negative to positive detuning into the same direction. The 3^2^P_3/2_ state enables the transitions to the 3^2^D_3/2_− and the 3^2^D_5/2_ state. Therefore not only the transition from 3^2^P_3/2_→3^2^D_5/2_ can be seen (main feature), but also the 3^2^P_3/2_→3^2^D_3/2_ transition. This is more faint, and can be observed at 1.47 GHz laser detuning above the main feature in the middle of the scan. This value corresponds to the (experimental) literature value of 1.469 GHz^33^. Others report slightly higher values^34^. To note that the slope differs from the other feature, and amounts to −1.53±0.08 GHz_819 nm_/GHz_589 nm_.

**Figure 4:**
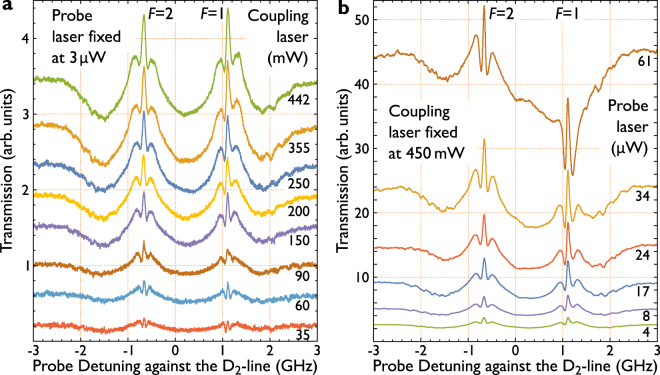
Power dependence. Power dependent measurements of the transparency signals. The signals shown are obtained by subtracting the background absorption spectrum (subtracting the referring spectra of Fig. 2). Examined is the dependency of the signals on the power ratio between probe and coupling laser. (**a**) Illustrates the dependency of the signals to the power of the coupling beam, while the probe beam is fixed to a power of 3 μW. Here a change of the width of all peaks and as well of all splittings between the peaks is observed; this holds for both features originating from *F* = 1 and *F* = 2. Subfigure (**b**) Shows the signal’s dependence on the power of the probe beam, while the coupling power is fixed to a power of 450 mW. Here no change of the width of the center peaks or the splitting between the side peaks is observed. The (green) signal in both measurements can be compared, they are measured at comparable powers. All the signals are normalized to the same value (maximum transmission of the green curve in (**a**)) and can hence be qualitatively compared in height. The peaks beside the main peaks form the anti crossings, visible in Fig. 3.

**Figure 5:**
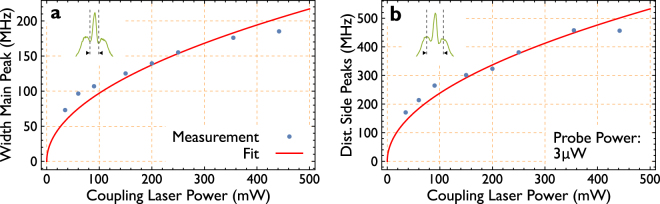
Power dependence fit. Evaluated distance of the peaks beside the main transparency peak which form the anti crossings (**a**) and the width of the main peak (**b**) in dependence of the power of the coupling laser. The data (blue) is obtained from the left set of peaks (*F* = 2) in Fig. 4. Fitted to the data is a root function illustrated in (red) and defined by Eqn 2.

An important measure is the power dependence of the observed effects. This would represent e.g. for EIT features an indirect measure of the Autler-Townes splitting. Figure 4a and b represent two measurements on the power dependence of the probe and the coupling laser. When the coupling laser power is increased, the relative strength of the signal changes. Furthermore, the width of the central peak and the splitting of the side peaks increases. A summary of the determined values is shown in Fig. 5.

For completeness, the probe laser power was changed. The shape of the signal is not directly affected, but pump effects become more prominent at the side of the initial observed features. Whereas the signal is comparable between the *F* = 1 and *F* = 2 states for lower probe powers, the features get asymmetric at higher probe powers. These slight deviations can also be observed in the 2 dimensional spectrum in Fig. 3b. To note, that this is again very crucial on the exact alignment of the overlap of the beams inside the cell.

Figure 5 shows the power dependent line width and splitting of the observed signal around the D_2_-line. The equation to fit the experimental results corresponds to a simple square root behavior. It can be observed that the distance between the peaks forming the anti-crossings and the width of the main transparency peak, qualitatively indicating that the Autler-Townes splitting (ATS) gets larger when the coupling power is increased. Also, the splitting of the peaks beside the main peaks increases.

In the dressed state picture the splitting between the dressed states is described by^5^

1 $$\hslash {\omega }_{\pm }=\frac{\hslash }{2}({{\rm{\Delta }}}_{{\rm{p}}}\pm \sqrt{{{\rm{\Delta }}}_{{\rm{p}}}^{2}+{{\Omega }}_{{\rm{p}}}^{2}+{{\Omega }}_{{\rm{c}}}^{2}})\,\mathrm{.}$$

Here, Δ_p_ denotes the detuning of the probe laser from the transitions and *Ω*_p_ and *Ω*_c_ are the respective Rabi frequencies of the probe and the coupling laser. In the resonant case of Δ_p_ = 0 and in the weak probe regime, where *Ω*_p_ ≪ *Ω*_c_ applies, this simplifies to $\hslash {\omega }_{\pm }=\frac{\hslash }{2}{{\Omega }}_{{\rm{c}}}$. This describes the spectral distance of a Autler-Townes doublet, but can also be approximately identified as the width of the transparency window of EIT. The width of the transparency window thus provides the value of the Rabi frequency of the coupling laser *Ω*_c_. Its width is then given as the Rabi frequency of the coupling laser *Ω*_c_ as

2 $${{\Omega }}_{{\rm{c}}}=d\sqrt{\frac{2}{cn{\varepsilon }_{0}{\hslash }^{2}A}}\sqrt{{P}_{{\rm{c}}}}\,\mathrm{.}$$

Here, *d* is the transition dipole moment of the respective transition, *c* is the vacuum speed of light, *n* is the refractive index, *ε*_0_ is the vacuum permittivity, $\hslash$ is the reduced Planck constant, and *A* is the cross sectional area of the laser beam.

Equation 2 is derived from the Rabi frequency ${\Omega }=({{E}}_{0}\cdot {d})/\hslash$. Further, a relation $I=P/A=$ $((c\cdot n\cdot {\varepsilon }_{0}\mathrm{)/2)|}{E}_{0}{|}^{2}$ is used to convert the electric field amplitude *E*_0_ to laser powers. The refractive index is approximated as *n* = 1. For the calculations of the intensity, the Gaussian shape of the beam is neglected and an uniform distribution of the laser power over its cross section is assumed, which is calculated to be 3.1 mm^2^ using the value of the beam waist as given above.

With this the transition dipole moment of the transition between the 3^2^P_3/2_ and the 3^2^D_5/2_ state (coupled by the coupling laser) is calculated to be approximately 2.1 × 10^−30^ Cm in the case of evaluating the width of the main transparency peak and approximately 5.1 × 10^−30^ Cm evaluating the data of the width between the side peaks. We like to remark that the calculations are for qualitatively understanding only and do not represent accurate values, especially in case of the splittings of the side peaks since they can actually not be related to the derived equations. The calculations have to be understood as efforts to evaluate the behavior of the signals to get information towards its origins. Figure 5 shows the evaluated data of the width of the main transparency peak and its side peaks of the D_2_-line, referring to *F* = 2. Despite the fact that the points deviate from the fit function, we find a value for the goodness of fit, the adjusted *R* ^2^-value as 0.9896 Fig. 5a and 0.9929 for Fig. 5b.

The literature value for the oscillator strength (or Einstein coefficient) *A*_ij_ for the transition 3^2^P_3/2_ to 3^2^D_5/2_ is ${A}_{3P\mathrm{(3/2)}-3D\mathrm{(5/2)}}=5.14\times {10}^{7}\,{s}^{-1}$ ^35^. This is converted into the reduced transition dipole moment using the equation^36^

3 $${{\rm{d}}}_{{\rm{ij}}}=\sqrt{{A}_{ij}\frac{3{\varepsilon }_{0}h{c}^{3}}{2{\omega }_{{\rm{ij}}}^{3}}}\,\mathrm{.}$$

This results in ${d}_{3P\mathrm{(3/2)}-3D\mathrm{(5/2)}}=3.2\times {10}^{-29}$ Cm for the reduced transition dipole moment of the transition, where this is an approximation and thus only gives the quantitative magnitude since the polarization and transitions between certain magnetic sub levels are neglected. For comparison, the transition dipole moment of the sodium D_2_ line is 2.9 × 10^–29^ Cm^30^.

A test for the origin of the effect, especially for higher lying Rydberg states, is the application of an electric field. For this, a new generation of the sodium vapor cells were equipped with electric field plates inside the cell. The plates were made of molybdenum, whereas the electrical throughput in the (borosilicate-)glass was made of tungsten. The field plates have a dimension of 80 × 10 mm^2^ and are separated by 6 mm. Utilizing 10 V applied voltage, the field results in a calculated value of 1666 V/m. Such electric fields are sufficient to suppress any Rydberg related signal in the cell, as confirmed by other experiments with Rydberg states in atomic sodium. This leads to a Stark shift of the atomic energy levels. Due to the high polarizability of the Rydberg states, these energy levels are affected much more than all lower energy levels. This implies that is would be possible to switch the EIT type signal on and off by switching the electric field. In the course of the described experiments, no further influence of an applied electric field was observed–a strong indication, that the desired Rydberg excitation was not yet achieved.

Another test for an efficient Rydberg excitation is the detection of other wavelengths in the fluorescence spectrum, when the atomic system decays via different pathways down to the ground state. For this, the cell was carefully thermally shielded, such that an optical access was possible from the side. Then, a 2*f* telescope was mounted on the side of the vapor cell, which guided the light from the excited region inside the cell by a multimode fiber to a grating spectrometer. For alignment, the hot cell and the telescope were supplied backwards to shine light into the vapor cell. The laser light was then set to be resonant to a sodium D line. The telescope was aligned such that the focus of the beam coming from the telescope overlaps with the beam passing the cell. Beside the dominant lines at 589 nm and at 819 nm, no further wavelength were detected. Other lines would indicate that additional decay channels were detected; this again indicates that the observed features do not depend on the two photon excitation of a Rydberg state.

## Discussion

The two-photon Rydberg excitation into the higher P states is particularly hard to achieve due to the required powers (≈kW/mm^2^, see e.g. Ref.^24^ to drive such a two photon transition. Still, with hundreds of mW optical powers in a sub-mm optical beam, we believe that such transitions might be observed. An intermediate level, as outlined above, can facilitate such an excitation nevertheless. The effective Rabi frequency is then calculated as^37^

4 $${{\Omega }}_{{\rm{eff}}}=\frac{|{{\Omega }}_{3{\rm{P}}-3{\rm{D}}}||{{\Omega }}_{3{\rm{D}}-n{\rm{P}}}|}{\mathrm{2|}{\rm{\Delta }}|}\,,$$

and depends on the Rabi frequencies of the transitions to and from the intermediate level *Ω*_3P–3D_, *Ω*_3D–*n*P_, and the detuning Δ. This detuning is given by the sum of the detunings of the two lasers Δ_3P–3D_ and Δ_3D–*n*P_ from the intermediate level divided by two: ${\rm{\Delta }}=({{\rm{\Delta }}}_{3{\rm{P}}-3{\rm{D}}}+{{\rm{\Delta }}}_{3{\rm{D}}-n{\rm{P}}}\mathrm{)/2}$. Where this is only valid for a certain two photon detuning of ${\delta }_{2{\rm{ph}}}=(|{{\Omega }}_{3{\rm{D}}-n{\rm{P}}}{|}^{2}-|{{\Omega }}_{3{\rm{P}}-3{\rm{D}}}{|}^{2}\mathrm{)/(4}{\rm{\Delta }})$ from the intermediate level. For this case perfect population transfer is achieved and can be adjusted by variation of the laser detunings. In the case where only one laser is used to drive both transitions, only the single detuning of this laser can be adjusted. Typically the detuning Δ has to be large enough i.e. $|{\rm{\Delta }}|\gg |{{\Omega }}_{3{\rm{P}}-3{\rm{D}}}|,|{{\Omega }}_{3{\rm{D}}-n{\rm{P}}}|$ in order to *not* populate the real intermediate state (3^2^D) to avoid problems due to spontaneous emission from this level^37^.

The exact transition energies into the higher Rydberg states have not been spectroscopically determined in the past. The feasibility of the suggested two photon process depends strongly on their exact values. By the quantum defects^38^ we have some estimation of the energy levels. A suitable level for a two photon excitation in case of exciting the D_1_-line is the Rydberg level with principal quantum number *n* = 117. The energy spacing between 3^2^P_1/2_ and 117^2^P is calculated to be 24433.42 cm^−1^ and hence approximately twice the spacing between 3^2^P_1/2_ and 3^2^D_3/2_, which is 12216.72 cm^−1^ ^29^. The detuning from the real intermediate level 3^2^D_3/2_ is thus approximately −211 MHz. Therefore, we assume that a Rydberg excitation by a two photon process might be achievable by the presented scheme from the D_1_-line. Still, we like to remark that even for allowed single photon transitions, such high Rydberg states are not commonly observed.

In case of exciting the sodium D_2_-line the Rydberg level with principal quantum number *n* = 52 is reasonable to be excited with the help of the intermediate level 3^2^D_3/2_, or 3^2^D_5/2_ The energy spacing between 3^2^P_5/2_ and 52^2^P is calculated to be 24399.60 cm^−1^. For the energy spacing between the levels 3^2^P_3/2_ and 3^2^D_5/2_ a value of 12199.80 cm^−1^ is given^29^. This results in a detuning in case of the two photon resonance from 3^2^P_3/2_ to 52^2^P of 9930.3 MHz from the real intermediate level 3^2^D_5/2_, and a detuning from the real intermediate level 3^2^D_3/2_ of 8431.4 MHz, according to a energy spacing between 3^2^P_3/2_ to 3^2^D_3/2_ of 12199.52 cm^−1^ ^27^. Subsequently, the envisioned two photon excitation from the D_2_ level seems to be harder to reach than from the D_1_ level.

One feature is the observed relative detuning behavior of the observed effect in the two density plots (Fig. 3). This is approximately the same for the features in case of the D_1_-line and the features belonging to the 3^2^D_5/2_ level in case of the D_2_-line. The features show a slope of approximately −2.08 GHz_819 nm_/GHz_589 nm_. The features of the D_2_-line belonging to the 3^2^D_3/2_ levels show a slope of approximately −1.53 GHz_819 nm_/GHz_589 nm_. For the “normal” ladder Rydberg EIT schemes^13,14,16^ this slope is estimated as

5 $$m=\frac{{\delta }_{{\rm{c}}}}{{\delta }_{{\rm{p}}}}=-\frac{{k}_{{\rm{c}}}v}{{k}_{{\rm{p}}}v}=-\frac{{k}_{{\rm{c}}}}{{k}_{{\rm{p}}}}=-\frac{{\lambda }_{{\rm{p}}}}{{\lambda }_{{\rm{c}}}}\,\mathrm{.}$$

In the presented case of the used wavelength of *λ*_p_ = 589 nm and *λ*_c _= 819 nm this would amount to a slope of −0.72 GHz_819 nm_/GHz_589 nm_. Thus the observed slopes of the main features are larger than the calculated one by a factor of approximately 3. This indicates that the process might be originating from an optical pumping process between the 3^2^P and the 3^2^D state, which is discussed below.

The aforementioned experiments exclude at the first sight the excitation of the desired, D state enhanced, S-P-(D)-P transition. This accounts for the experiments with the spectrometer, where no other higher Rydberg decay channels are observed. Further, a good fraction of the Rydberg population would be strongly influenced by an electric field. To note, that it still might be that the observed effects dominate the spectrum, and spurious transfer into the Rydberg states is not or only weakly observed. So far, no further more sensitive experimental approaches, such as e.g. lock-in detection schemes have been attempted.

One problem to efficiently excite the Rydberg P states might be their small transition probability. Other authors did not observe higher order transitions into the P-Rydberg states originating from the 3_2_D states^39,40^. Instead, the Rydberg P states are significantly weaker than the reported F states^40^, which are nicely resolved in ionization experiments.

For a theoretical description, we compared the experimental results with calculations with a Lindblad master equation. To include all four levels (S-P-D-P), the derived equation includes the Hamiltonian and the Lindblad super-operator, both being 4×4 matrices. To determine the resulting spectrum all velocity classes were integrated. The procedure is equivalent to the usual math applied to such problems^16^. The observed slope of −2.08 GHz_819 nm_/GHz_589 nm_ is not predicted, as it is observed in the experiment.

We like to remark, that a comparable experiment on the same wavelength was performed earlier by other authors^41^. The main difference is the directionality of the utilized beams. Their results are described in terms of sum frequency mixing. There, the detection is also changed to a side-wise detection of 342 nm light (3^2^D → 3^2^S), which is formed as a sum frequency mixing of 589 nm and 819 nm. The authors describe two relevant processes: a) the two-step process. b) the two-photon excitation.

The described scaling behavior is $2\times {{\Omega }}_{3{\rm{S}}-3{\rm{P}}}{}^{3}\times {{\Omega }}_{3{\rm{P}}-3{\rm{D}}}\,$ for the two step process, and $\,{{\Omega }}_{3{\rm{S}}-3{\rm{P}}}\,\times {{\Omega }}_{3{\rm{P}}-3{\rm{D}}}\,$ for the two photon process. This description of the relevant transitions strength suggests a dependence of the different features on the Rabi frequency of the probe field. This behavior is indicated in our data in Fig. 4. When the probe laser power is increased, the relative strength of the inner feature gets more prominent than the outer, broader feature. As outlined in the literature^41^, this coincides with the more narrow two step process, which is outlined in the other paper.

When Rydberg excitations are excluded, the observed effects originate likely from another level interplay. Only the levels 3^2^S → 3^2^P and 3^2^P → 3^2^D seem to be involved. A closer look on the two dimensional D_1_ spectrum (Fig. 3a), shows the changing strength between the two lines with negative and positive detuning. This suggests an optical pumping effect between the *F*′ = 1 and *F*′ = 2 levels. This implies, that when midway between the two 3^2^P levels, both lines should be equal–a situation given at zero detuning (seen in Fig. 2 and 3). When the pumping is below or above this value, one line should be dominating due to this pumping effect into the other state. When the detuning dependence (slope of the dominant line) from one laser to the other laser is in-cooperated, the slope of −2.08 GHz_819 nm_/GHz _589 nm_ can be explained by a three photon process is suggested. This is an interplay between the P and D state with three (equivalent) photons, described in the literature^42^. One option to research on the involved levels and the pumping effect would be to utilize another laser system, which can depopulate the 3^2^P state. Such experiments were performed in the past with atomic rubidium and are described as multi-wave mixing^43^.

## Conclusion and Outlook

Two dimensional spectroscopy of hot atomic sodium vapor was shown on the 3^2^S → 3^2^P and the 3^2^P → 3 ^2^D transitions. The experimental concept to implement an (allowed) two photon transition between two P states in atomic sodium is presented. The technicalities of such an experiment would be easier than for usual Rydberg EIT excitations. Further, this would enable a variety of Doppler-free spectroscopic tools, such as for electromagnetically induced transparency.

Simple spectroscopic measurements, such as observations on a spectrometer and the influence of an electric field reveal that likely only the levels 3^2^S, 3^2^P and 3^2^D are involved. Other lines are not experimentally observed here. The 2D-detuning dependence, and the change of the relative intensities of the involved 3^2^P transitions, suggests the combination of the D-line transition and 3 photons, resonant around the 3^2^ P → 3^2^D transition.

Although that we did not find any direct evidence of driving a three photon transition, we are confident that such a transition can be driven in a Doppler-free configuration. Ideally, an intermediate level (such as the 3^2^D level in the presented study) can achievement of a reasonable Rabi frequency to drive such a transition. Further studies, with e.g. lock-in detection might be able to detect a fingerprint of such a transfer. In such experiments the coupling laser can be modulated and the modulation signal can be detected on the probe beam detector. This would correspond to an effective modulation transfer spectroscopy^31^. In our spectroscopic investigations, the absence of detected higher level decays which would indicate a transfer to a higher Rydberg state can also be supported by more efficient filtering the detected light with an atomic medium^44^.

In terms of an atomic quantum memory, the introduced scheme for the S → P and the P → D transition has been recently investigated in atomic cesium^45^. It is suitable for a short-term storage of quantum information, limited by the motional dephasing of the utilized atomic vapor cell. Furthermore the *T*_1_-lifetime of the excited 6^2^D state will be limiting the usability of the memory for long-term quantum storage. How far the presented 3^2^S → 3^2^P → 3^2^D transitions in atomic sodium can be used for a comparable quantum storage scheme is presently under research. As a convenient single photon source, a single organic dye molecule which is resonant to the 3^2^S → 3^2^P transition will then be utilized as a probe field^10,46^.
